# Malaria, Intestinal Helminths and Other Risk Factors for Stillbirth in Ghana

**DOI:** 10.1155/2010/350763

**Published:** 2010-04-01

**Authors:** Nelly J. Yatich, Ellen Funkhouser, John E. Ehiri, Tsiri Agbenyega, Jonathan K. Stiles, Julian C. Rayner, Archer Turpin, William O. Ellis, Yi Jiang, Jonathan H. Williams, Evans Afriyie-Gwayu, Timothy Phillips, Pauline E. Jolly

**Affiliations:** ^1^Department of Epidemiology, School of Public Health, University of Alabama at Birmingham (UAB), Birmingham, AL 35294-0022, USA; ^2^Division of Preventive Medicine, School of Medicine, University of Alabama at Birmingham (UAB), Birmingham, AL 35294-0022, USA; ^3^Mel and Enid Zuckerman Collage of Public Health University of Arizona 1295 N. Martin Avenue A256, Tucson, AZ, USA; ^4^School of Medical Sciences, Kwame Nkrumah University of Science and Technology, Kumasi, Ghana; ^5^Department of Microbiology, Biochemistry & Immunology, Morehouse School of Medicine, Atlanta, GA 30310, USA; ^6^Division of Infectious Diseases, School of Medicine, University of Alabama at Birmingham (UAB), Birmingham, AL 35294-0022, USA; ^7^Komfo Anokye Teaching Hospital, Kumasi, Ghana; ^8^Department of Biochemistry, Kwame Nkrumah University of Science and Technology, Kumasi, Ghana; ^9^College of Agriculture and Environmental Sciences, University of Georgia, Griffin, GA 30602, USA; ^10^Department of Veterinary Integrative Biosciences, Texas A&M University, College Station, TX 77843, USA

## Abstract

*Objective*. The objective of the study was to assess Plasmodium/intestinal helminth infection in pregnancy and other risk factors for stillbirth in Ghana. *Methods*. A cross-sectional study of women presenting for delivery in two hospitals was conducted during November-December 2006. Data collected included sociodemographic information, medical and obstetric histories, and anthropometric measures. Laboratory investigations for the presence of *Plasmodium falciparum* and intestinal helminths, and tests for hemoglobin levels were also performed. *Results*. The stillbirth rate was relatively high in this population (5%). Most of the stillbirths were fresh and 24% were macerated. When compared to women with no malaria, women with malaria had increased risk of stillbirth (OR = 1.9, 95% CI = 1.2–9.3). Other factors associated with stillbirth were severe anemia, low serum folate concentration, past induced abortion, and history of stillbirth. *Conclusion*. The fact that most of the stillbirths were fresh suggests that higher quality intrapartum care could reduce stillbirth rates.

## 1. Introduction

Of the 130 million babies born worldwide every year, approximately 4 million are stillborn [1], more than 98% of these occur in developing countries [2]. Stillbirth accounts for more than half of perinatal mortality in developing countries [3]. In Sub-Saharan Africa, stillbirths account for more than 3% of deliveries each year [2]. While countries in South-East Asia report the highest overall numbers of stillbirth, countries in Africa report the highest incidence rates per 1000 live births [4]. The average stillbirth rate in developing countries has been reported to be 26 per 1000 live births, about five times higher than in developed countries (5 per 1000) [4]. One fourth to one third of all stillbirths is estimated to take place during delivery [5, 6]. Stillbirths occurring in the intrapartum period generally have a normal appearance and are often called “fresh” stillbirths [5]. The skin not being intact implies death more than 24 hours before delivery (antepartum), often called “macerated” stillbirths [5].

 Stillbirths have not been widely studied, have been under-reported, and rarely have been considered in attempts to improve birth outcomes in developing countries [5, 6]. There are many factors associated with stillbirth including inadequate access to obstetric care, inadequate care [7], malaria, hypertensive disease, poor nutritional status, history of stillbirth, congenital anomalies, sickle cell disease, and high burden of infectious comorbidities [5, 8–10]. 

 Conceptually, infection may result in fetal death through several pathways [11]. First, maternal infection may cause severe illness, leading to fetal death [12, 13]. Also, an infection in the uterus or anywhere else in the mother's body may precipitate preterm labor [14]. Last, the placenta may be directly infected, leading to reduced blood flow to the fetus, a likely cause of stillbirth associated with malaria infection [15]. When malaria parasites infect the placenta, placental insufficiency results because of lymphocyte and macrophage accumulation, and increased expression of pro-inflammatory cytokines; these impede maternal blood flow through the placenta [16, 17]. Intestinal helminths, including hookworms and *Trichuris trichura,* have been associated with anemia [18, 19]. A study in Tanzania showed that 63% of stillbirths were attributable to maternal anemia [20]. It has been suggested that low hemoglobin concentrations can cause a state of chronic hypoxia, which is presumably exacerbated in pregnancy when oxygen demands are particularly high because of the metabolism of the mother and the fetus, and that oxygen transfer to the fetus is probably reduced in anemic women [21]. Folate deficiency causes megaloblastic anemia [22]. Circulating folate concentrations decline in pregnant women, hence the need for supplementation [22]. A strong association has been observed between maternal plasma, cord plasma, and placental folate concentrations, suggesting that transplacental folate delivery depends on maternal plasma folate concentrations [22]. 

 According to the World Health Organization's Opportunities for Africa's Newborns 2006 report, the stillbirth rate for Ghana is 24 per 1000 deliveries. Even though stillbirths represent a large proportion of perinatal deaths, causes of stillbirths are poorly understood in Ghana. To our knowledge, the association between malaria and intestinal helminth coinfection in pregnancy and stillbirth has not been studied. Few studies have studied the association between malaria and helminths in pregnancy, with conflicting results. This study provides baseline data in this area. Given that 98% of stillbirths occur in developing countries, especially sub-Saharan Africa [2], which also has a high burden of malaria and intestinal helminth [23] infections, it is important to investigate the role of these infections in contributing to stillbirth.

## 2. Methods

The study was conducted in Kumasi, the capital of the Ashanti region of Ghana. Kumasi is the second largest city in Ghana with a population of 1.2 million [24]. The climate in Kumasi is humid and tropical, with two rainy seasons, April to June and September to October. Helminth infection is endemic in the Ashanti region [25], which also has an intense perennial malaria transmission, the predominant parasite being *Plasmodium falciparum* [24]. 

 The Institutional Review Board of the University of Alabama at Birmingham and the Committee on Human Research, Publications and Ethics, School of Medical Sciences, Kwame Nkrumah University of Science and Technology, Kumasi reviewed and approved the study protocol before its implementation.

 A cross-sectional study of women presenting for delivery at two hospitals in Kumasi, the Komfo Anokye Teaching Hospital (KATH), and the Manhyia Polyclinic was conducted in November and December 2006. All women with a singleton, uncomplicated pregnancy were asked to participate. After informed consent was obtained, a questionnaire was administered to collect sociodemographic information, and medical and obstetric histories. Body weight and mid upper arm circumference (MUAC) were measured for each woman. Obstetric information was also obtained from the mothers' antenatal care (ANC) charts. ANC charts provided information on number of antenatal care visits, gestational age as assessed by palpation at first ANC visit or ultrasound at first ANC, tetanus shots, malaria prophylaxis, antihelminth medications, hemoglobin level, and illnesses and treatments during pregnancy. Blood was drawn by venipuncutre for determination of hemoglobin levels, serum folate level, and malaria antigen tests. Stool samples were obtained for determination of intestinal helminths.

At delivery, state of the newborn (alive or stillbirth), sex, weight, and length were obtained as recorded by the midwives. 

 Determination of malaria antigen in plasma was done using the Malaria Antigen Celisa (Cellabs, Brookvale, Australia). The malaria Antigen Celisa kit is a monoclonal antibody-based assay specific for *P. falciparum *malaria. The assay detects a merozoite antigen that circulates in the blood for up to 14 days postinfection. Determination of Hookworms, *Ascaris lumbricoides, *and *Trichuris trichura* was done using the Kato-Katz thick smear technique (WHO, 1991). Stool samples were processed within 12 hours of collection and examined microscopically within one hour of preparation to avoid missing hookworm ova. For *Strongyloides stercoralis*, samples were processed using the Baermann method [26]. Serum folate was measured by radioimmunoassay. Hemoglobin level was measured in an automatic cell counter (Sysmex M-2000; Digitana AG, Hamburg, Germany) about 30 minutes after blood sampling.

 Variables were defined as follows—*uncomplicated pregnancy*: absence of hypertension, pre-eclampsia, history of a previous caesarean section and hemorrhage, and a normal presentation of the fetus [27]. *Malaria infection*: presence of malaria antigen in the mother's peripheral blood at the time of delivery. *Intestinal helminth infection*: presence of helminth eggs or larvae in stool collected at the time of delivery. *Coinfected*: positive for both malaria and intestinal helminths at delivery. *Anemia*: hemoglobin levels <11 g/dL of blood, and *severe anemia*: hemoglobin level <8 g/dL [28]. *Low serum folate*: serum folate concentration <6.8 nmol/L [29]. *Stillbirth*: an intrauterine death of a fetus weighing at least 500 grams after 20 completed weeks of gestation occurring before the complete expulsion or extraction from its mother [30]. An intrauterine death of a fetus during labor or delivery was considered a fresh stillbirth, and an intrauterine death of a fetus sometime before the onset of labor, where the fetus showed degenerative changes was considered a macerated stillbirth [30]. *Induced Abortion*: the purposeful interruption of an intrauterine pregnancy with the intention other than to produce a live-born infant, and which does not result in a live birth [31]. 

 Sample size was calculated using unpublished reports on stillbirth from the two study hospitals, which estimated that at least 1%–1.5% of 1000 births would be stillbirths. We made the assumption that if we obtained 10 stillbirths, and that 10–25% of women with normal births had both malaria and intestinal helminth infections, at a 5% significance level, we would have 80% power to detect an odds ratio of 7.5–9.0; assuming 15 stillbirths, we would be able to detect an odds ratio of 6.0–7.5.

## 3. Data Analysis

Data analysis was performed using SAS software version 9.1 (SAS Institute, Cary, NC). Differences in socio-demographic and obstetric characteristics by stillbirth were assessed by chi-square or *t*-test. Correlation analyses were performed to identify potential multicollinearity between independent variables. To determine factors associated with stillbirth, we used multiple logistic regression. Variables that were significant (*P* < .05) on bivariate analysis and those that are known to be associated with stillbirth based on previous studies were entered into a regression model [32]. Through this procedure, we calculated odds ratios (OR) and 95% confidence intervals (CI).

## 4. Results

Seven hundred and eighty five (785) women were recruited into the study before delivery in the two hospitals in Kumasi. We obtained both malaria and intestinal helminth results from 746 women, and data analysis was limited to these women. None of the women smoked and only 14 (1.8%) consumed alcohol. Overall, the mean age of the women was 26.8 years (range: 15 to 48 years); 21.1% were single, 30.2% were primigravidae, 30.6% were anemic, 29.5% had a prior induced abortion, and 5.2% had a history of stillbirth (Table 1). 

 There were 37 cases of stillbirths (4.9% of all deliveries). Of these, 9 (24.3%) were macerated. A higher proportion of women who were single did not receive SP during pregnancy, had fewer than 5 ANC visits, had low folate levels, were anemic, had had a prior induced abortion or a prior stillbirth and delivered a stillborn infant compared to their counterparts (Table 1). 

 Of the 746 women, 407 (54.6%) had neither infection, 147 (19.7%) were infected with *P. falciparum* only, 68 (9.1%) were infected with helminths only, while 124 (16.6%) were coinfected. A higher proportion of women with either organism presented with stillbirth than women with neither infection. Women who were coinfected had a modestly higher rate of stillbirth than women with a single infection (Table 2). 

 Low serum folate, severe anemia, prior induced abortion and prior stillbirth were each strongly, independently associated with stillbirth, with increased odds ranging from over 3-fold to a 6-fold increase (Table 3). Women with malaria irrespective of whether or not they had intestinal helminths had a 90% increased odds of stillbirth. Although intestinal helminth infection had a stronger association, it was not statistically significant (Table 3).

## 5. Discussion

This study demonstrated that the study population had a relatively high rate of stillbirth (5% of all deliveries). Factors associated with stillbirth were malaria, severe anemia, low serum folate concentration, past induced abortion, and history of stillbirth.

Many stillbirths were fresh (75.7%), an indication that a proportion of these cases could likely have been prevented [5]. 

 It has been suggested that stillbirths occurring in the peripartum period could be prevented through appropriate cesarean section, improved obstetric care, and improved emergency response to obstetric complications [5]. In this study, women who had fewer antenatal care visits had an increased risk of stillbirth, suggesting that stillbirths are closely linked to use and quality of maternal services [33]. 

 Malaria is endemic in many African countries, and is thought to play a role in contributing to stillbirth [9]. We observed an association between malaria and stillbirth. A similar finding has been observed in sub-Saharan Africa [34]. Intestinal helminths, especially Hookworms and *Trichuris* can cause anemia [18, 19], which in turn leads to adverse birth outcomes including stillbirth [20]. We did not observe an association between intestinal helminths and stillbirth, a finding that has been previously reported [35]. However, our observation could be the result of small numbers, that is, malaria was more common than intestinal helminthes. Coinfection with malaria and intestinal helminths did not increase the risk for stillbirth but as in the case of intestinal helminths, this could be a matter of numbers. A study in Tanzania found that 63% of stillbirths were attributable to anemia [20]. Malaria contributes to anemia by hemolysis or destruction of parasitized cells and causes shortened red cell survival [36, 37], while hookworms and Trichuris cause anemia through direct blood loss [19, 38]. Since the mechanisms by which malaria and intestinal helminth infections cause anemia differ, it is possible that their impact on anemia are additive [39] and could exacerbate adverse birth outcomes. Anemia was a risk factor for stillbirth in this study. The association between anemia and stillbirth has been demonstrated previously [20]. Low serum folate was associated with stillbirth. Folate deficiency causes megaloblastic anemia [22]. Circulating folate concentrations decline in pregnant women, hence the need for folate supplementation [22]. A strong association has been observed between maternal plasma, cord plasma, and placental folate concentrations, suggesting that transplacental folate delivery depends on maternal plasma folate concentrations [22]. Some studies [40, 41] have reported higher rates of stillbirth in women with megaloblastic anemia than those without.

 Another risk factor for stillbirth in our study is history of induced abortion. Abortion is legal in Ghana only for medical reasons, and is not available upon request (Ministry of Health, Ghana). Most women seeking abortion therefore sometimes attempt illegal abortions, and then go to the hospital for treatment of complications [42]. Removal of retained products of conception in the hospital setting is usually performed by cervical dilation and curettage [43]. There has been concern that this may result in cervical insufficiency, hence future adverse birth outcomes [44].

 History of stillbirth substantially increased the risk of stillbirth in the study population. The tendency to repeat pregnancy outcomes in successive births is well known and includes risk of stillbirth [45]. Previous studies have demonstrated that women with a history of stillbirth may have a 6 to 10-fold increased risk of stillbirth [46, 47]. The causal mechanism may involve impaired placental development and function due to compromised vascular support system [48].

 A methodological weakness of our study lies in limited power due to the number of stillbirths, therefore our findings should be interpreted with caution. The fact that it was a cross-sectional study also limits our ability to draw causal or temporal associations. The study however has several strengths including new findings, high participation and consistency with other studies in some risk factors of stillbirth, which strengthens confidence in the new findings. Another strength of the study lies in the fact that the hospitals in which the study was conducted are secondary and tertiary hospitals which cater to large numbers of women of all socioeconomic status from Kumasi and surrounding areas. The 2008 Demographic Health Survey for Ghana (DHS, 2008) reported that 82.4% of women in urban areas in Ghana deliver in a health facility. Our results are therefore representative of most pregnant women in the area. 

 The fact that most of the stillbirths were fresh suggests that higher quality intrapartum care could reduce stillbirth rates. More studies need to be conducted to further assess the association between stillbirth and malaria and intestinal helminth coinfection. It is important to conduct further studies to investigate risk factors of stillbirth to determine which stillbirths are preventable so that targeted interventions can be developed and tailored for resource-poor settings.

## Figures and Tables

**Table 1:** Demographic and obstetric characteristics of Ghanaian women by stillbirth status, 2006 (*N* = 746).

			Stillbirth				
	ALL		No (*N* = 709)		Yes (*N* = 37)		
Characteristics	*N*	%	*N*	%	Yes	%	*P*-value
Age:							
<20	102	13.7	96	13.5	6	16.2	.95
20–24	188	25.2	178	25.1	10	27.0	
25–29	215	28.8	205	28.9	10	27.0	
≥30	241	32.3	230	32.4	11	29.7	
Formal education							
None	164	22.1	157	22.2	7	20.0	.16
Primary	98	13.2	89	12.6	9	25.7	
Middle or Junior Secondary	363	48.9	348	49.2	15	42.9	
≥Senior Secondary	117	15.8	113	16.0	4	11.4	
Weekly income							
<100,000	175	23.7	162	23.0	13	38.2	.21
100,000–199,000	49	6.6	48	6.8	1	2.9	
200,000–354,000	295	39.9	284	40.3	11	32.4	
≥355,000	220	29.8	211	29.9	9	26.5	
Marital Status							
Single	156	21.1	143	20.3	13	37.1	.05
Living in union	140	18.9	134	19.0	6	17.1	
Married	445	60.1	429	60.8	16	45.7	
Gravidity							
One	225	30.2	216	30.5	9	24.3	.19
Two	141	18.9	137	19.3	4	10.8	
≥Three	380	50.9	356	50.2	24	64.9	
Trimester at first ANC visit							
First	389	52.8	370	52.5	19	54.3	.72
Second	325	43.9	311	44.1	14	40.0	
Third/none	23	3.5	24	3.4	2	5.7	
Less than 5 ANC visits	318	43.2	296	42.2	22	62.9	**.02**
Sulfadoxine pyrimethamine doses							
None	197	26.4	177	25.0	20	54.1	<**.01**
One	196	26.7	188	26.5	8	21.6	
Two	99	13.3	94	13.3	5	13.5	
Three	254	34.1	250	35.3	4	10.8	
No deworming	719	96.9	685	96.9	34	97.1	.93
Folate level							
Low	290	55.6	262	53.5	28	87.5	<**.01**
Normal	232	44.4	228	46.5	4	12.5	
Hemoglobin level							
Normal (≥11 g/dL)	512	69.5	496	70.8	16	44.4	<**.01**
Moderate anemia (8–10.9 g/dL)	192	26.1	179	25.5	13	36.1	
Severe anemia (<8 g/dL)	33	4.5	26	3.7	7	19.4	
Previous induced abortion	217	29.5	192	27.5	25	65.6	<**.01**
History of stillbirth	27	5.2	16	3.3	11	39.3	<**.01**

Numbers in each category may be less than the total due to missing values; Bold *P* < .05; *N* = number.

**Table 2:** Malaria and intestinal helminth infection status of 746 Ghanaian women according to whether or not they had stillbirths, 2006.

	ALL		Stillbirth	
Infection Status	*N*	col %	*N*	row %
Malaria				
yes*	271	36.3	22	8.1
no	475	63.7	15	3.1
*P* * =*				**<.01**
Helminths				
yes*	192	25.7	17	8.9
no	554	74.3	20	3.6
*P* * =*				**<.01**
Uninfected	407	54.6	10	2.5
Malaria alone	147	19.7	10	6.8
Helminths alone	68	9.1	5	7.4
Malaria and helminth coinfected	124	16.6	12	9.7
*P* * =*				**<.01**

Col = column; *N* = number; *with or without other infection. Bold *P* < .05.

**Table 3:** Risk factors associated with stillbirth in Ghana, 2006.

Characteristics	Crude	^(**a**)^Adjusted		^(**b**)^Adjusted	
	OR	OR	95% CI	OR	95% CI
Age (per 5 years)	**1.4**	**1.6**	1.2–2.3	**1.2**	1.1–1.8
Single	**2.3**	0.9	0.1–6.7	0.8	0.1–5.8
Primigravidae	0.7	N/A		N/A	
No SP doses	**2.5**	2.7	0.8–9.3	2.3	0.9–13.3
First trimester ANC visit	1.0	**2.2**	1.2–10.2	2.9	0.7–11.9
Low serum folate	**3.6**	**3.9**	2.0–16.2	**3.5**	1.9–17.1
Anemia					
Moderate versus normal	**2.3**	3.3	0.9–11.3	2.9	0.7–11.8
Severe versus normal	**4.8**	**4.2**	2.7–38.9	**4.3**	2.8–41.8
Past induced abortion	**5.3**	**3.6**	2.2–22.6	**3.8**	2.4–26.5
Past stillbirth	**5.7**	**6.4**	3.8–31.2	**6.1**	3.6–33.1
Infection status					
Malaria (yes versus no)	**2.7**	**1.9**	1.2–9.3	N/A	
Intestinal helminths (yes versus no)	**2.6**	2.1	1.0–14.1	N/A	
Infection status					
Malaria only versus uninfected	**2.9**	N/A		1.7	0.4–8.7
Helminths only versus uninfected	**3.2**	N/A		2.8	0.6–19.5
Coinfected versus uninfected	**4.3**	N/A		1.7	1.0–9.7

OR-Odds Ratio. CI- Confidence interval. Bold *P* < .05.

^(a)^Model includes 2 individual infections with or without the other infection.

^(b)^Model includes single infections and coinfection.
